# Lectures on the Operations of Surgery and on Diseases and Accidents Requiring Operations

**Published:** 1846-10

**Authors:** 


*Lectures on the Operations of Surgery and on Diseases and Accidents requiring operations.* By ROBERT LISTON, ESQ., F. R. S. *Senior Surgeon to the University College Hospital, and Professor of Clinical Surgery in the College. With numerous additions, by* THOMAS D. MUTTER, M. D., *Prof. of Surgery in Jefferson Medical College, Philadelphia, etc., etc.* 1 vol. 8vo. Lea & Blanchard Publishers, 1846.

We have first of all a little private spleen to vent upon our friend Dr. Mutter; a penny worth of complaint, which we have been treasuring and nursing for some days past, and we must not loose this most favorable opportunity to be rid of it. Our complaint runs thus — two years since we were promised a complete work upon Surgery by Dr. Mutter himself; and knowing the Doctor's responsibility, we cleared up a niche in our library and filed away the promise, regarding it as good as a two pound note on the Bank of England. We have waited thus long in confident and daily expectation of its full payment, never doubting that it would be met with principal and every cent of interest brought up to date. But mark our disappointment! We have at the expiration of the two years, Liston's Lectures with *notes by Mutter!* Now we appeal to our brethren whether this is strict justice — are these *notes* by Mutter a full and honest payment of the old note? Does it not amount to "repudiation"? We shall not deny the value of the payment received, and we consent to endorse a six per cent. interest, but we refuse positively to give up the paper.

Before we open the book, we have one thing more to say. These *nineteen Lectures* were given by Mr. Liston before the students of the London University, and constituted but the opening of his course. And as each successive lecture was delivered, it was stenographically stolen and published in the Lancet. Now here is a "case" for those English literary despots, those book-worm aristocrats, those justice-seeking, type-protecting, eternally-prating "international-copy-right-law-leaguers," who charge us with defrauding them of their just revenue, because we sell their thoughts "done up in a cheap way" full three thousand miles from Pater Noster Row. But herein Mr. Wakely has been guilty of piracy within the very doors of the University! How shall he answer when he shall appear before his judges? Mr. Liston had published already two books, and it were easy to prove, by the plainest arithmetic, that he intended to publish a third — nay, perchance a fourth — and thus we might figure his losses — "two thousand ducats in that, and no satisfaction, no revenge," and then too it is the choicest book of the three — in these lectures he had treasured all his new and most precious inventions — as the "art of holding a knife," and the true philosophy of "sharpening a razor," with many other valuable and curious secrets for surgeons and barbers, and which he "would not have exchanged for a wilderness of monkeys." Are not these just grounds of complaint on the part of Mr. Liston — that he has been robbed, and his children have been robbed, and "Tom," the feline protege, who always sat at his patron's table, and whose tender palate will feel the earliest approach of economy in the *cuisine?* And has twenty years wrought such a change in the laws of England, that the self same Lancet which was forbidden to "bleed" Mr. Abernethy, is now permitted to "let blood" from Mr. Liston and Sir Benjamin, both gentlemen of the Royal Household, and no Lord High Chancellor interferes, with his "injunctions" and charges of "literary frauds," and "breaches of trust," to save them from absolute syncope?

Away with all this carping about an international copyright law to protect *English* literature, when the English recognise no rights of property at home. We care not how soon the "stamp act" is imposed. The interest of this country has long reqired some high tariff, to save us from foreign manufactures; but of one thing we must assure our trans-atlantic friends — when we cannot sell their books as "cheap literature" we shall not sell them at all. We shall always be willing to pay them cost of paper and printing, but the "oil" with "wear and tear of mind" must be thrown in.

*Hemorrhage.*—Of 39 pages upon this subject, more than 37 are from the pen of Dr. Mutter. Mr. Liston never writes biographies of dead operations, but he is a straight forward, practical man, who speaks but little and to the purpose. Hemorrhage, says Mr. Liston, may be arrested by compression or by ligature. A small, round, thread ligature, tied lightly. You shall not stop to secure vessels until an operation is completed; since to do so is troublesome, tedious and unnecessary. You are not to rely upon the new fashioned, crazy practice of torsion; it may answer in a few small vessels, but it is better to tie them.

All our readers are familiar with Mr. Liston's mode of *closing* and *dressing wounds.* He still recommends his favorite isinglass plaster and water dressing. Dr. Mutter uses the water *cold* in winter and *warm* in summer, first approximating the edges of the wound, whenever it is proper, and then laying upon the surface moist pledgets of patent lint; every hour also, an assisstant should pour upon the lint a table-spoonful or two of water.

In the second lecture Mr. Liston treats of *injuries* of the *scalp* and *skull,* and the following remarks thrown out incidentally, in relation to blood letting, are sufficiently curious in these days, to attract attention.

"The lancet, I believe, is an instrument that I use less than any other; nevertheless, I can tell you, possibly, something of its use and abuse. Bleeding may often be required, but it is, no doubt, often resorted to unnecessarily and improperly, and many patients are thrown into a bad state of health, or even die in consequence. Death may occur from the too frequent and too great abstraction of the vital fluid, and it not unfrequently happens from the local effects of injury to the vein. All of these risks, however, must of necessity be incurred in many severe cases of inflammatory action. But think twice, and well, before you resort to this expedient; the same good effects often enough follow less hazardous means." p.72.

The lecture is wound up by a recapituation of the circumstances under which it may be proper to apply the trephine.

1. To allow matter collected under the cranium to escape, and blood, if you will, compressing the brain; 2, to remove splinters of bone in cases of compound fracture; 3, in order to relieve the symptoms of an oppressed brain when a portion of bone is lying much beneath its proper level and causing deep coma and other serious syptoms of compressions; 4, to remove foreign bodies." p.86.

We have been in the habit of stating our rule for the use of the trephine in an *adult* thus. 1, we may not trephine for a mere fissure where the proper signs of compression are absent; 2, we may not trephine for a fracture with depression of bone where there exists no external wound communicating with the fracture, and the signs of compression are absent; 3, we are never to employ the trephine where the levator or Heys-saw can be substituted. But we may operate in any case in which the signs of compression are decided and persistent, whether it be a simple fissure, or a fracture with depression without external wound, or with external wound; we may operate for compression produced by extravasation of blood or by formation of pus, provided always we can discover any reasonable indications of the situation of the blood or pus, and provided the depot can be reached by the trephine. We may trephine adults also for epilepsy in certain cases. Again, in *children*, not over 3 years of age, we seldom trephine, or operate otherwise, for the purpose of elevating the bones, even when the bones are depressed and the *symptoms of compression are marked;* and not unless a wound already exists through which the elevator can reach the depressed bones, or unless the signs of compression (coma, &c.,) have continued undiminished during several hours.

*Operations on the eye.*—Small encysted tumors often occur on the lids, especially upon the upper lid ; sometimes they are found in the very substance of the cartilage. It is a thin cyst containing a glairy fluid. Do not attempt to dissect them out, or you will make a hole through the lid. Evert the lid and open the cyst by a single incision from the inside, push out the contents, lacerate the cavity a little, and leave it. It discharges a few days and then is well. Now and then it returns, and then you will open it again in the same manner, and after the bleeding has ceased, you may introduce carefully into the sac a minute drop of nitric acid, or a minute portion of caustic potass — the latter requires much caution.

After *scarifying the lids*, (for which leeches are now generally substituted,) if you would promote the bleeding, you must keep the lids everted, and continue to wipe away the blood as it flows, with a small soft sponge, or a piece of lint.—p. 95.

The following remarks on the subject of *Pterygium*, by Dr. Mutter, are just.

"I have occasionally seen much trouble in cases of pterygium from a free dissection of the membrane. In consequence of the large surface exposed, cicatrization takes place with considerable *contraction*, and in one or two cases I have seen an incurable squint from this cause alone produced. My attention was first directed to this point by my excel ent friend, Dr. Thos. Harris, surgeon-in-chief of the navy. To avoid this difficulty, I usually cut out a large piece from the centre of the membrane, and then cauterize the edges of the wound with nitrate of silver. Treat it as you will, however, this is often a most vexatious complaint, returning over and over again, and of course requiring repeated operations."

*Strabismus*, "about which so much noise has been made of late." Mr. Liston, as we think properly enough, discountenances M. Guerin's subcutaneous method of operating. He advises also, never to cut more than one muscle in any one eye, and never to carry the dissection beyond the complete division of the muscle. A speculum, scissors, forceps and blunt probe, are all the inrtruments required. (We prefer the dog-tooth forceps to the spring forceps, used by Liston, and never use a speculum.) After the operation, cover the *sound* eye for twenty-four or forty-eight hours, etc. Mr. Liston has seen two or three cases where the eye has been lost from the violence of the inflammation succeeding the operation !

p. 108–9. Prof. Mutter remarks upon the general results of the operation as gathered by M. Velpeau, that if "not more than one half" have been relieved, "it is probable that many of the cases were unfit for the operation," and that in his own practice, "at least two thirds have been perfectly cured, while a larger proportion have been materially relieved." We shall be permitted also to add, that in our own operations, a careful resume shows of sixty-eight cases, forty-eight perfectly successful, sixteen imperfect, and four totally unsuccessful. In one case the vision of the eye operated upon has been entirely lost, in consequence, as we think, of the change of the axis of vision, but in no case has a serious inflammation occurred. We have *seldom* cut both eyes in the same patient, and in only one instance, (and then we had operated upon both eyes,) have we produced to the slightest extent, an opposite divergence. We therefore continue to regard this operation, where properly made, and the cases are properly chosen, as one of the happiest surgical achievements of the age.

*Fistula Lacrymalis* This is an affection sufficiently troublesome, as every surgeon can attest, and every suggestion of experience must be received with attention. "Some cases are irremediable," the bony canal itself being completely obliterated. Slight cases are relieved by a mild astringent collyrium, or by leeching, a blister about the size of a finger nail over the sac, or by painting the part with a strong solution of iodine. When these fail an attempt may first be made to probe the nasal duct from below, but in some cases the passage is too irritable. It is of no use, Mr. Liston thinks, to inject the sac from the puncta, or to introduce small probes. (We certainly have succeeded in several cases of long standing in relieving for months the epiphora, and in a few cases in effecting a permanent cure, by the careful and repeated introduction of slender probes from the puncta into the nostril. The probes should not, however, in any case be allowed to remain in the passage longer than one hour at a time, and then only when inflammation does not exist. The probe must be flexible, and after it is fairly introduced, it must be bent so as to assume as completely as possible the course of the canal. This plan cannot, however, by any means be generally relied upon. If an actual *fistula* exists, you are not to attempt to follow the tract of the fistula into the sac, but make a new opening opposite the sac and introduce the common style. The plan recommended by Dupuytren, of introducing a *tube*, and allowing the artificial opening to close over it, is not entitled to confidence; nor can Mr. Liston sanction the old practice of penetrating the os unguis into the middle meatus, where the nasal duct is found impervious. The style may after a while be removed during the day and worn only at night. How long its use may be necessary is indeterminable.

*Cataract.* A valuable chapter of about 30 pages, entirely by Dr. Mutter. The practical surgeon will distinguish the Doctor from a mere speculative writer, by the following judicious remark.

"By far the most simple, most easy of execution, and at the same time most useful in the majority of cases, is an operation intended to destroy cataract by exposing it to the action of the aqueous humour."

A brief lesson which the universal advocates for extraction would do well to remember, and which, had Baron Wenzel been earlier taught, a "hat full of eyes" might have been saved to his unfortunate patients.

We shall continue our review of this work in the next number of the Journal. F. H. H.

